# Efficacy/Safety of the Use of Glucocorticoids in Oral and Maxillofacial Surgery

**DOI:** 10.3390/dj11100239

**Published:** 2023-10-17

**Authors:** Heilyn Joanna Nils, Cristina Arce Recatala, Antonio Castano, David Ribas, Javier Flores-Fraile

**Affiliations:** 1 University of Salamanca, 37008 Salamanca, Spain; j.flores@usal.es; 2 Valencia International University, 46002 Valencia, Spain; cristina.arce@campusviu.es; 3 University of Sevilla, 41004 Sevilla, Spain; acastano@us.es (A.C.); dribas@us.es (D.R.)

**Keywords:** corticosteroids, anti-inflammatory postoperative oral surgery, dexamethasone, dentistry, dosage form design, route of administration, cervicofacial infection

## Abstract

Introduction: Glucocorticoids, also known as corticosteroids or steroids, are drugs derived from cholesterol. They are synthesized by the adrenal cortex, along with other hormones, such as cortisol and aldosterone. Glucocorticoids are drugs recommended for patients undergoing surgery on the oral cavity, facial skeleton, and related cervical structures due to their high efficacy against inflammatory and immune processes. However, these drugs are restricted due to their multiple and serious adverse effects. The objective of this study was to verify the efficacy of corticosteroids administered in major surgeries of the oral cavity, as well as of the cervical and facial structures, based on the characteristics of the patient so as to select the best therapeutic strategy. Methods: Articles in the databases of PubMed, Nature Portfolio, Medline, Cochrane Library, and Google Scholar were thoroughly examined. Results: A total of 54 articles were selected to address the proposed objectives. The results obtained show that it is effective and safe to use glucocorticoids as pre- or postsurgical therapy in oral and maxillofacial surgery to control the processes of inflammation, pain, lockjaw, and edema. However, when referring to the use of these drugs, one must proceed with caution and pay particular attention when handling them. The concentration of the glucocorticoids used must be individualized, as well as the selection of the route of administration. Various studies show that, although the oral route is the most used route, the most effective route is the intramuscular route due to its easy absorption. However, for patients who have recurrent inflammatory and vesiculobullous ulcerative lesions, the topical route should be chosen to mitigate side effects, considering that recurrent applications must be made to prevent the worsening of the lesion and to avoid having to use medications enterally. In patients with cervicofacial infections, antibiotics continue to be the main drugs used to manage the condition in conjunction with corticosteroids. It is important to know the possible interactions of glucocorticoids with other medicines or food: it has been described that the interaction between Ritonavir, an antiretroviral drug that inhibits human immunodeficiency virus (HIV) proteases, and prednisone causes an increase in the concentration of prednisone, leading to possible toxicity in normally safe doses and, in many cases, iatrogenic Cushing’s syndrome. It is also important to know the systemic or topical adverse effects of the chronic or high-dose use of glucocorticoids. Conclusions: It can be concluded that by making adequate use of glucocorticoid therapy in oral and maxillofacial surgery to manage clinical manifestations, it is possible to attenuate the morbidities of treatment and intervention.

## 1. Introduction

Steroids are the most used medication, with high efficacy against inflammatory and immune diseases, pain, edema, and lockjaw [1]. Glucocorticoids are drugs derived from cholesterol (hence their name as steroidal drugs). They are synthesized by the adrenal cortex, along with other hormones, such as cortisol and aldosterone. Synthetic glucocorticoids are classified according to several criteria: according to the duration of action (short duration, 8–12 h; intermediate duration, 12–36 h; and long duration, more than 36 h); according to their glucocorticoid potential compared to the endogenous glucocorticoid hydrocortisone; and according to their mineralocorticoid activity or ability to mimic the mineralocorticoid effects of aldosterone, promoting water and sodium retention and favoring potassium elimination [2].

When describing the mechanism of action of these drugs, it must be stated that the adrenal cortex oversees the synthesis of glucocorticoids, cortisol, hydrocortisone, and corticosterone, hormones that are released throughout the day following a circadian rhythm and that have numerous effects at metabolic, cardiovascular, nervous system levels, etc. These steroid hormones exert their mineralocorticoid or glucocorticoid function through binding to two types of nuclear receptors: the glucocorticoid receptor (GR or type II in the old nomenclature) and the mineralocorticoid receptor (MR or type I). These act on different genes, producing changes (stimulation or inhibition) in protein synthesis and tissue response. The performance of these corticosteroids is regulated by the hypothalamus-pituitary-adrenal cortex axis. Thus, the hypothalamic corticotropin-releasing hormone prompts the production of pituitary adrenocorticotropin, or ACTH, which then promotes cortisol output at the adrenal level. When cortisol levels are sufficient, and to avoid the excessive accumulation of cortisol, negative feedback to the axis occurs such that the release of CRH, ACTH, and cortisol is interrupted. Corticosteroids are analogs of endogenous glucocorticoids, and they prevent the entry of leukocytes into the inflammatory focus, disturb the action of fibroblasts and endothelial cells, and mitigate the performance or effects of numerous chemical mediators of inflammation [1,3].

As for the pharmacological effects of these drugs, it can be pointed out that they have anti-inflammatory actions, which are achieved through multiple mechanisms that include the inhibition of histamine release and consequent capillary vasodilation, the inhibition of prostaglandin synthesis, the inhibition of proinflammatory cytokines (COX2, PLA2, INOS IL, and TNF), and the increased synthesis of anti-inflammatory cytokines and annexin (which causes apoptosis). With regard to immunosuppressive actions, through a lymphocytic effect, T lymphocytes reduce the production of IL-2 and IFNϒ, preventing the activation of Tc lymphocytes and natural killer (NK) cells. B lymphocytes, by contrast, are relatively resistant to the effects of glucocorticoids: they inhibit their proliferation only if they are administered before they are activated. At small doses, they do not affect the production of antibodies, but at high doses, an increase in catabolism and a slight decrease in synthesis are observed, perhaps due to indirect mechanisms [4,5].

The use of glucocorticoids is restricted due to their multiple and severe adverse effects. In turn, they are also very powerful antiallergens indicated in allergic processes, such as rhinitis, contact dermatitis, situations that occur with hives—and even in the treatment of anaphylactic shock once the patient has been stabilized with adrenaline—and skin diseases (psoriasis and atopic dermatitis, among others). Due to their immunosuppressive properties, they are also used in therapy for autoimmune diseases (e.g., systemic lupus erythematosus, psoriasis, and rheumatoid arthritis) and in the prevention of transplant rejection. They are also used in states of steroid deficiency, such as Addison’s disease, a disorder that manifests itself when the body does not produce enough of certain hormones secreted by the adrenal glands [6].

The adverse effects of glucocorticoids are a prolongation of their effects and include (a) intense muscle atrophy; (b) hyperglycemia, which can condition the appearance of diabetes mellitus, the appearance of cataracts, osteoporosis, peptic ulcer, delayed wound healing, and increased susceptibility to infectious processes; (c) iatrogenic Cushing’s syndrome—abnormal fat redistribution and fat accumulation in the abdominal region; and (d) the suppression of adrenal function, since it decreases with hypothalamus-pituitary-adrenal cortex axis activity, and a decrease in the production of endogenous glucocorticoids. It should be considered that the adrenal cortex atrophies in a period of 2–3 months, after which treatment cannot be interrupted abruptly because it would lead to the appearance of adrenal insufficiency, which could be fatal. To address this, glucocorticoid doses are slowly decreased over weeks and even months [5]. The objective of this article is to review the efficacy of the administration of glucocorticoids using the various routes of administration and the appropriate doses of each drug.

Focus question: presented in the “PICO” format.

What is the effectiveness/safety of the use of glucocorticoids in patients undergoing oral and maxillofacial surgery without previous pathologies?

**Hypothesis:**  *The use of corticosteroids is more effective (but not safe) in alleviating inflammation and pain processes in oral and maxillofacial surgery than the use of NSAIDs.*

### 1.1. General Objective

The objective of this paper is to study the effectiveness/safety of the use of glucocorticoids in oral and maxillofacial surgery in young and adult patients without other previous pathologies.

### 1.2. Specific Objectives

1. Identify the applications of glucocorticoids in oral and maxillofacial surgery in adolescents and adults.
2. Determine the appropriate dose to be administered in oral and maxillofacial surgery in adolescent and adult patients where there is no presence of other previous pathologies.
3. Examine the advantages and disadvantages of the different pharmaceutical forms of glucocorticoids.
4. Highlight the interactions of glucocorticoids with other drugs.
5. Describe the effectiveness when glucocorticoids are administered to attend oral manifestations and maxillofacial diseases in adults (aged 16–65 years).
6. Study the adverse reactions of glucocorticoids after prolonged use in patients undergoing oral and maxillofacial surgery.

## 2. Methodology

### 2.1. Review Type

This work is a systematic review, and it was carried out following the PRISMA declaration (Preferred Reporting Item for Systematic Reviews and Meta-Analysis) and the criteria for the publication of a systematic review. Registration number is: CRD42023445218. The aim was to gather the information available in different databases to respond to the proposed objectives. A search was carried out in the period between 7 April 2023 and 2 June 2023.

### 2.2. Search Strategy

**Figure 1:**
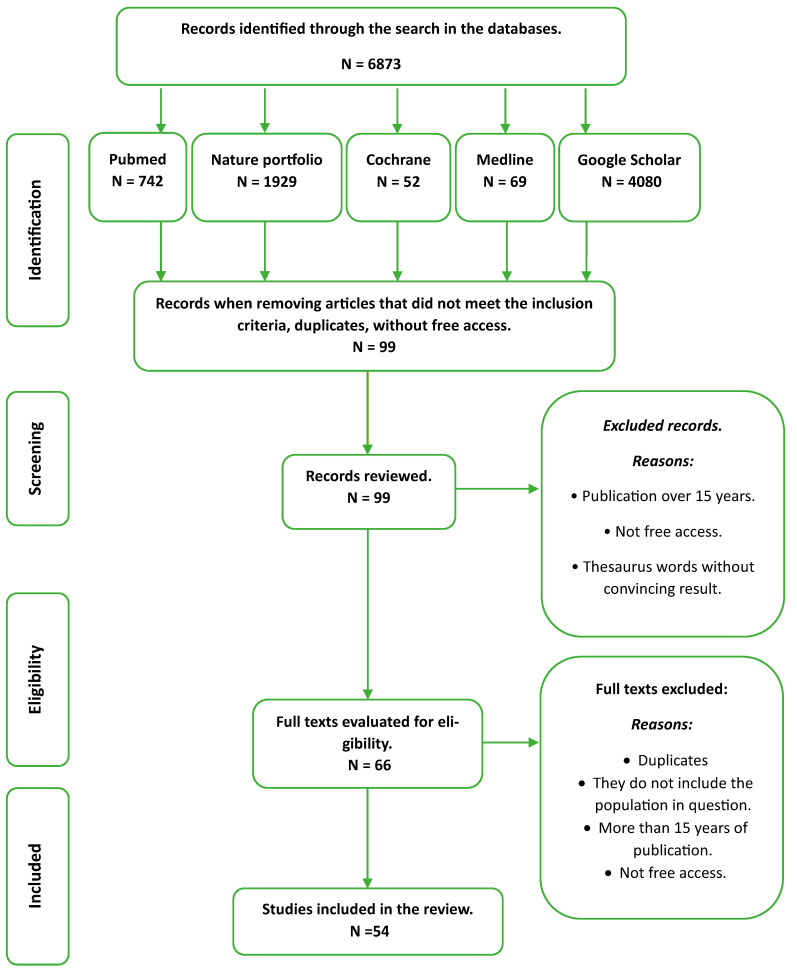
Scheme of the methodology using a flowchart of identified, excluded and included studies.

Information was searched for in the PubMed, Nature Portfolio, Cochrane, Medline, and Google Scholar databases, using free terms, as well as MeSH terms, in the indicated cases and combining them with the Boolean AND and OR operators. During the search, thesaurus keywords limited to the English language were used, such as corticosteroids, anti-inflammatory postoperative oral surgery, dexamethasone, and dentistry. Likewise, parentheses were used to specify search combinations, and quotation marks were used to carry out searches with terms containing multiple words. In addition, books were examined. Finally, publication date filters were used, limiting the search so that it included articles published in the last 10–15 years (Figure 1).

In the PubMed database, 742 results were obtained; when using the filters focusing on the last 10–15 years, 51 articles were obtained, of which 20 were selected.

In the Nature Portfolio database, 1929 results were obtained. When using filters that covered the inclusion criteria, this was reduced to 9 articles, of which 3 were selected.

In the same way, 52 articles were obtained from the Cochrane database, and 69 results were obtained from Medline. The search of these databases was carried out by applying the filters already mentioned and by only looking for articles that responded to the proposed objectives; 1 article was chosen from each database.

Finally, searches were carried out for the journals with the greatest impact in the Google Scholar search engine, with the most recent randomized clinical trials to sustain the vast study carried out on the subject in question. With our initial approach, 4080 articles were obtained, and by using filters that covered each inclusion criterion and the relevant dates, searching only in English, Portuguese, and Spanish, and rejecting those that were found to be duplicates, this was reduced to 37 articles, of which 27 (21 in English and 6 in Spanish) were useful in contributing information to this review.

### 2.3. Selection Criteria

To carry out the systematic review adequately and accurately, selection criteria were applied (Table 1).

### 2.4. Study Selection and Quality Assessment

#### Bias Analysis

The main biases that must be considered during the search of databases are as follows:

✓ Publication bias must be considered, specifically in studies whose results do not coincide with the prescribed interval.
✓ The compilation of the data must be systematic and homogeneous; when there is heterogeneity in the obtained results, it hinders the combination and comparison when different populations are studied.
✓ High heterogeneity may affect the validity of the results of the meta-analysis if included in this study, e.g., a difference between the age of the patients in each population and the results obtained.

To assess the risk of bias in this systematic review, each piece of data must conform to the objectives set for the review. We used the Cochrane risk of bias tool to assess the methodologies of the examined studies, please see (Table 2).

## 3. Results

**Figure 2:**
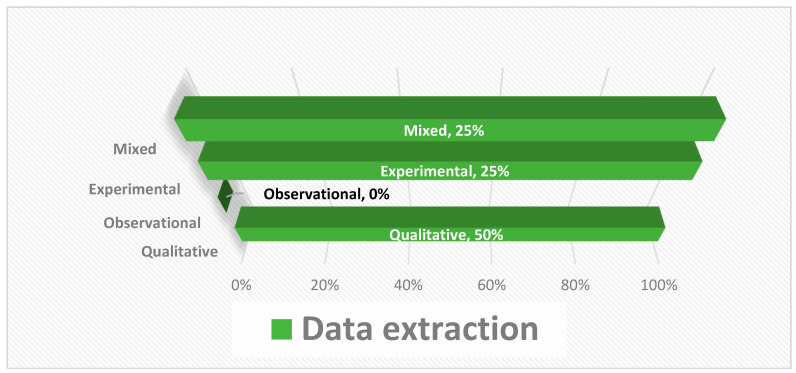
Results of data extraction.

Of the total number of studies analyzed, 50% were qualitative, 0% were observational, 25% were experimental, and 25% were studies with mixed methodologies (quantitative and qualitative). The main results of each of the reviewed articles are shown below (Figure 2).

1. Identify the applications of glucocorticoids in oral and maxillofacial surgery in adolescents and adults (Table 3).

Currently, the vast therapeutic application of glucocorticoids, such as betamethasone, dexamethasone, methylprednisolone, triamcinolone, or prednisolone (resulting in the greater use of glucocorticoids and little to no use of mineralocorticoids), provides great benefits in acute and chronic conditions or ailments that have an allergic, inflammatory, or immunological basis [12,13].

* Temporomandibular joint disorders (TMJs): Preparations with ester (acetonide) that are insoluble in water release gradually and, therefore, have a longer effect. Formulations without ester (sodium phosphate) that are soluble in water release more quickly, and, therefore, their effect is shorter; such preparations include betamethasone sodium phosphate and dexamethasone [13,14,15].

* Oral lichen planus (OLP): Here, medium-potency topical corticosteroids are usually used; high-potency fluorinated drugs and, recently, halogenated corticosteroids have been incorporated due to their high potential. The great disadvantage and, therefore, inconvenience that results from using these topical medications is insufficient adherence to the mucosa. For recalcitrant erosive lichen planus erythematosus, where topical drug application is not effective, a more powerful systemic medication is used: prednisolone between 15 and 30 mg or until it reaches 35 mg between 5 and 7 days. Likewise, the injectable application of triamcinolone 10–20 mg 2–4 x/week for 2 weeks is also used [16,17,18,19].

* Bell’s palsy: This inflammation of the unknown cause of the facial nerve can be effectively treated by administering prednisolone 1 mg/kg/day, with a maximum dose of 60–80 mg for 7–10 days during the first week and a decrease in the dose in the following week. This protocol is carried out in patients with a normal immune response [20,21].

Oral Ulcerative and Vesiculobullous Lesions

* Recurrent aphthous stomatitis (RAS): Canker sores derive from a non-infectious inflammation process. Topical and systemic steroids seek to limit the inflammatory worsening of this condition. Denture patients can mix steroids with an adhesive such as orabase. For more extensive lesions, a gauze with the steroid can be placed on the ulcer and left to act for 15 to 30 min. For lesions in areas of accessibility or with a more complex visualization, a topical dexamethasone elixir of 0.5 mg is administered, applying it with a gauze on the pustules 4×/day for 15 mts. In the presence of major thrush, administering prednisone systemically is recommended with an initial therapeutic approach of 40 mg/day with a duration of one week to fight the outbreak, gradually decreasing the dose after 7–14 days have elapsed [22,23].

* Behçet’s disease: Particularly manifested as a mucocutaneous condition with the appearance of oro-genital ulcers and skin lesions, this disease can be effectively managed with anti-inflammatory agents that modify the action of neutrophils; treatment is based on immunosuppressive therapy [24].

* Pemphigus diseases: There are two main groups that characterize these autoimmune blistering disorders: “The pemphigus diseases” and “The autoimmune blistering diseases of pemphigus”. In turn, we can classify them into three main subtypes: “Vulgar pemphigus”, “Foliaceous pemphigus”, and “Para neoplastic pemphigus”. For the pattern of pemphigus vulgaris, it is essential that therapy is based on systemic immunosuppression, choosing drugs such as corticosteroids. If there is resistance to improvement, the dose can be adjusted and increased up to 2 mg/kg/day.

When topical therapy is used, the purpose of which is to alleviate inflammation and prevent secondary infections, calcineurin inhibitor corticosteroids, either combined or not combined with antibiotics, are administered. It should be noted that, when treating oral lesions, gels containing local anesthetics and appropriate care with a dental health professional should be incorporated [25,26,27].

For pemphigus foliaceus, topical doses between the range of 10 and 40 mg (one tube per day) have proven to be feasible for the use of prednisolone systemically. In seriously ill patients, systemic therapy should be used, with the corticosteroid of choice being prednisone at 0.5 mg/kg/day. This dose can be decreased 4–6 months after the initial treatment. The adjustment of this dose occurs at the start of maintenance therapy [28,29]. Finally, for neoplastic para pemphigus, glucocorticoids at a dose of 1–2 mg/kg/day are the quintessential treatment of choice due to their efficacy [18].

* Erythema multiforme: Patients can use a topical treatment; clobetasol propionate mouthwashes in an aqueous solution provide good management due to the contact time between the lesion and the drug. For patients who are not systemically involved, moderate-to-severe (ME) cases can be treated with systemic glucocorticoids. In patients with severe lesions, prednisone is recommended at 40–80 mg/kg/day for 1–2 weeks and then a rapid decrease in its dose. It is worth mentioning that prednisone at a dose of 0.5–1.0 mg/kg/day as initial systemic therapy or pulses of methylprednisolone at 1 mg/kg/day for 3 days have shown efficacy in the event of injury [30].

* Postherpetic neuralgia: Recently, researchers have suggested that the subsequent treatment to be followed should be based on a topical therapy of lidocaine or capsaicin and a systemic therapy based on gabapentin, pregabalin, and tricyclic antidepressants [31]. It is highly effective to use 40 mg methylprednisolone injections for 10 days, highlighting that there is great fear regarding its safety due to the possible risk of arachnoiditis or fungal meningitis [32].

* Central giant-cell granuloma (CGCG): There is still no complete understanding of its mechanism of action. Researchers have stated that administering an intralesional steroid injection will cause fibrosis and reossification of the lesion in the bone cyst due to the impediment of the excretion of lysosomal proteases, which inhibit osteoclasts and promote their apoptosis. The habitual treatment is surgical removal [33]. However, regarding the choice of corticosteroid, dexamethasone is recommended, by which, in order to obtain an optimal response, the patient must be disciplined in administering injections two or three times a week without interruption for three consecutive months [34].

* Other pathologies:

It is of great importance to refer to patients who present cervicofacial infections, where the use of corticosteroids or lack thereof could be beneficial. A brief review of studies carried out in the United Kingdom is presented below.

Studies show that, given the high number of mortalities caused by cervicofacial infections, great interest has been generated in the use of steroids as adjuvants to reduce the serious effects of these conditions. These infections arise through different pathways, such as pharyngeal tonsils, teeth, salivary glands, esophageal trauma, and necrotic lymph nodes. However, they manifest in a similar way. Among them, we can mention peritonsillar abscess (PTA), periorbital cellulitis and abscess, pharyngitis, epiglottitis, supraglottitis, and DNSI (deep neck space infection).

Usually, patients are subjected to medical and surgical treatments (intravenous broad-spectrum antibiotics, or, when feasible, an incision is made, followed by drainage and aspiration), but what must be highlighted about this review is that, by incorporating corticosteroids as coadjutants, they minimize airway edema, lockjaw, and pain, simultaneously causing adverse effects, such as exacerbating infection and weakening the immune response, among other powerful complications in patients with possible systemic conditions or who are immunocompromised, thus creating controversy because of these unwanted manifestations [35].

The vast majority of studies suggest the following high doses for a short period of time, all administered intravenously (IV) [35]:

Hydrocortisone at 200 to 1050 mg/daily;

Dexamethasone at 8 to 10 mg/7 days;

Methylprednisolone at 1 to 3 mg/kg/5–7 days (parapharyngeal abscess (IV); epiglottitis (IV); supraglottitis (IV).

2. Determine the appropriate dose to be administered in oral and maxillofacial surgery in adolescent and adult patients where there is or is not a manifestation of previous pathologies.

Referring to the proper dosage when using corticosteroids, several factors must be considered: the doses, which must be specific to each patient depending on the pharmacokinetics of the different formulas; the possible presence of pathology or alteration that is suffered in the area of the body to be treated; the possible interactions of the drugs with non-steroidal agents and concomitantly administered steroids; and the patient’s response to treatment [36].

Various mechanisms are involved in the suppression of inflammation by corticosteroids, such as blocking the inflammatory process at an early stage or, in situations where the process is already well established, providing rapid resolution in favor of eliminating inflammation, highlighting the classification of the corticosteroids mentioned above.

Below is a breakdown of the appropriate doses of the steroids used in oral and maxillofacial surgery, focusing on glucocorticoids, which are the preferred alternative (those with an intermediate effect and that are long-acting) to mineralocorticoids (Table 4) [37].

Brief effect: Hydrocortisone and cortisone. Intermediate effect: Prednisone, prednisolone, methylprednisolone, and triamcinolone. Prolonged activity: Betamethasone and dexamethasone.

3. Examine the advantages and disadvantages of the different pharmaceutical forms of glucocorticoids.

It is vitally important to have clear knowledge and be competent as health professionals when choosing to use glucocorticoids as postsurgical therapy. Understanding the advantages and limitations of their different routes of administration can aid decision making since the scarcity of information regarding the different pharmaceutical forms that can be used can result in a decrease in the effectiveness of the therapy, an increase in pharmacological cost, and even malpractice.

Below are details of the different advantages and disadvantages of the routes of administration:

The enteral route involves the administration of the drug through the digestive tract. In turn, it is classified into different subtypes that include the oral route, the sublingual route, and the rectal route [38].

The oral route is the most physiological way through the mouth (swallowing, drinking, and chewing). However, it has a slow start of action (approximately 45–60 mts, not being useful for emergency situations). Other drawbacks are that the patient must be willing to take the medication and that they must be able to swallow. In addition, it must be considered that drugs, once absorbed in the intestine, access the liver, where they can undergo the hepatic first-pass effect, which can destroy and inactivate a certain amount of the drugs. The first-pass effect consists of hepatic metabolization before the drug exerts its therapeutic effect [39].

The alternative is to choose to treat chronic conditions. Drugs transit through the liver filter, where those that require it are reduced and become biologically active. They are adequately absorbed through the gastrointestinal tract and usually take 30 min to enter the circulation [40].

Corticosteroids that are taken orally are likely to have the highest likelihood of generating side effects according to the dose administered. Among their disadvantages are manifestations such as fluid accumulation (lower limbs), hypertension, psychological effects (memory, confusion, and delirium), and weight gain [41]. Patients exposed for a longer time may present vision problems (glaucoma or cataracts); high blood sugar; an increased risk of bacterial, viral, and fungal infections; bone fractures and bone wear (osteoporosis); muscle weakness; bruising; and slower wound healing [42].

In the sublingual route, a tablet is placed under the tongue, resulting in a very rapid absorption of 5–10 min (highly vascularized area). For example, in the case of a heart attack, nitroglycerin is administered sublingually. Its function is to relax the vessels. The drug passes directly into the blood, thus avoiding the hepatic first-pass effect. This route is recommended to achieve the rapid therapeutic action of drugs that cannot be administered orally. One drawback is that this mucosa is exclusively permeable to the passage of highly lipid-soluble substances [38,43].

The administration of drugs via the rectum has the advantages of being suitable for use in patients with vomiting and for drugs that may be too irritating to the stomach. This route does not require the willingness of the patient, although it is an uncomfortable route, so it could cause embarrassment. It has a high absorption, but it is irregular [44].

## 4. Parenteral Route

The parenteral route includes the following [45]:

The intravenous (IV) route allows direct access into the bloodstream. A characteristic of this pathway is that it has no absorption. Through this route of administration, the fastest action is achieved (approximately 1 min).

The intramuscular (IM) route is where the drug is injected deep into the muscle tissue, which is highly vascularized tissue. The rich vascularization of the muscle provides rapid absorption (10–30 min) (faster than the oral route). As a drawback, it should be mentioned that, when using this route, constant administration at the same site can cause irritation and local fibrosis, with a progressive reduction in absorption; therefore, it is necessary to vary the injection point.

The subcutaneous (SC) route is where the drug is delivered into the subcutaneous/adipose tissue. The blood flow in this tissue is much less than in the muscle; therefore, the absorption is slower than that in the IM route (widely used for the administration of insulins, heparins, and some vaccines).

The topical route involves the application of the drug to the skin and mucous membranes. This is used when looking for an additional form of administration. Depending on the part of the body where the medication is administered/deposited, we have the transdermal route, inhalation route (inhalers/nebulizers), nasal route, auditive route, ocular route, vaginal route, or rectal route, with the drug traveling through the corresponding mucous membranes. This route of administration should not be confused with the transdermal route (the route of administration through the skin). A systemic effect is usually sought over the entire organism, as occurs when fentanyl transdermal patches are administered for the treatment of severe pain. Triamcinolone acetate vs. triamcinolone is the most effective [45].

The intra-articular route provides differing levels of absorption. If the objective is to achieve a local effect, drugs must be partially insoluble to reduce systemic absorption, and the concentrations in the intra-articular space are high. Usually, the corticosteroids used are hydrocortisone acetate, prednisolone tebutate, and triamcinolone hexacetonide [45].

It should be noted that this route of administration comes with warnings, as the continuous use of dexamethasone can cause Cushing’s syndrome [46]. In 1974, Hooley and Hohl concluded that the topical use of steroids prevents ulceration and excoriation or the chafing that results from the reduction in tissue on the lips or corners of the mouth [47].

4. Interactions of Glucocorticoids with Other Medications

Dexamethasone and, to some degree, prednisolone are both absorbed by hepatic cytochrome P450 3A4 (CYP3A4); therefore, when addressing the interaction of steroids with other drugs, the expectation is that CPY3A4 inhibitors increase the systemic response to glucocorticoid [48].

In more than one study, it was shown that Ritonavir is linked to iatrogenic Cushing’s syndrome and symptomatic GI-AI when combined with intranasal corticosteroids such as Fluticasone [49,50].

When co-administering Ritonavir (a drug used to treat infection by the virus that causes AIDS) and prednisone, disseminated prednisolone concentrations increased by up to 37%. The newer protease inhibitor dolutegravir (a drug used to decrease the amount of HIV in the blood and boost the immune system) does not restrain CYP3A4 and could be an option in patients who have been given steroids [50]. It is not recommended to receive treatment via inhalation, intranasal, or injectable routes of administration with the simultaneous administration of budesonide, Fluticasone, and triamcinolone with CYP3A4 due to their metabolism. If necessary, beclomethasone and flunisolide may be reliable options [51].

It is particularly important to highlight this since HIV-infected patients are more likely to develop asthma and obstructive pulmonary disease [52].

Other interactions are shown below (Table 5) [3]:

5. Describe the effectiveness when glucocorticoids are administered to attend oral manifestations and maxillofacial diseases in adults (16–65 years).

### 4.1. Population Study in the Range of 18 to 30 Years

* De la Cruz Carranza et al. [8]. In 2013, they conducted a study to compare the effectiveness of prophylactic dexamethasone at 8 mg with that at 4 mg. Oral medications were used to control the postsurgical edema of third molars. Edema causes discomfort and interrupts the daily work of patients who undergo the extraction of third molars.

Very few studies show the use of different doses and routes of dexamethasone to treat the postsurgical edema of impacted third molars, and they were carried out in populations with different genetic characteristics. The introduction of evidence-based therapeutic protocols is needed, which will benefit clinicians and patients. In this randomized parallel clinical trial, a population with an age range between 18 and 30 years participated, and it was recorded that the group that received dexamethasone at 8 mg orally presented greater efficacy in the control of postsurgical edema, which leads us to say that dexamethasone has been shown to be a drug safe for administration, with an adequate duration and dose. This result coincides with that which was found by Filho et al. [8]. At a higher dose of the drug, there is a higher concentration in plasma and, consequently, greater anti-inflammatory activity [8].

### 4.2. Study of Population over 21 Years

* Manríquez-Guzmán et al. [9]. In 2013, they took samples from 116 patients between 21 and 45 years of age. They aimed to make a comparison between patients who presented severe acute inflammation and were medicated either before or after the extraction of the lower third molars. Within the actions of glucocorticoids, early and late phenomena of inflammation must be inhibited (they inhibit phospholipase A2, and, according to recent studies, they inhibit the expression of the enzyme cyclooxygenase 2 (COX-2) and attenuate the immune response (the production of antibodies can be reduced by excessive amounts of glucocorticoids)). Severe acute inflammation, as well as the consumption of drugs, was higher in the population that did not receive glucocorticoids prior to the intervention [9].

* Bhandage et al. [7]. In 2018, they evaluated the roles of the intraoperative administration of hydrocortisone and postoperative dexamethasone in reducing postoperative complications after major surgeries of the oral cavity performed under the effect of general anesthesia. They decided to choose 20 patients in a population with an age range between 25 and 65. On the second day postsurgery, an average pain reduction of 70% was noted, and on the fourth day, an overwhelming reduction of 97% was noted. An overall 12 mm reduction in edema was observed over the course of the four-day hospital stay. This result led to the conclusion that the administration of a single intraoperative dose of hydrocortisone and an adjusted postoperative dose of dexamethasone helps combat most postoperative complications and, therefore, contributes to the healing of the surgical site. It is known that, following procedures carried out in the oral cavity, the surgical site tends to become contaminated due to the existence of saliva, bacteria, and contaminants from the stomach flora through acid reflux and postoperative events such as vomiting [7].

### 4.3. Population Study in the Range of 16 to 35 Years

* Núñez-Díaz et al. [11]. In 2019, a trial was carried out on 60 patients with an age range from 16 to 35. Divided equally into two groups, 4 mg of dexamethasone was randomly applied intramuscularly to the pre-surgical and postsurgical groups. Facial edema was evaluated through the distance between facial points, lockjaw was evaluated through the interincisal distance, and pain intensity was evaluated through the numerical scale (EN).

It was concluded that the values of facial edema were reduced in group A at 60 min compared to those in group B. Regarding lockjaw, there was an insignificant difference between both populations during the evaluations carried out in relation to pain, with the highest peak being felt at six hours in both groups. This study highlighted that the pre-surgical administration of dexamethasone showed a substantially higher decrease in facial edema after extraction of the third molar of the lower jaw [11].

### 4.4. Population Study in the Range of 18 to 25 Years

* Chávez-Rimache et al. [10]. In 2020, they carried out a randomized trial involving 54 patients in a population between 18 and 25 years of age. This study demonstrated that, according to the VAS (visual analog scale), adjuvant therapy with group B vitamins (B1, B6, and B12) considerably increased the analgesic effect of dexamethasone at 3, 6, 12, 24, and 48 h after surgery (third molar, lower jaw).

In the lower jaw (third molar) surgery, the preoperative intramuscular use of dexamethasone with group B vitamins showed a representative increase associated with analgesic activity and a significant decrease in total analgesic consumption compared to the preoperative intramuscular administration of dexamethasone alone. The swelling had a similar behavior in both study groups, with no significant difference being found.

The preoperative administration of corticosteroids is effective in delaying and preventing postoperative sequelae since the tissue therapeutic level of the drug is present from the onset of the inflammatory response. Dexamethasone is a long-acting corticosteroid that has a synergistic effect with NSAIDs; however, the main problem is the presence of adverse effects, such as nausea and the increased risk of gastrointestinal bleeding. The intramuscular route of administration is the one of choice, as it has proven to be more effective in reducing pain and postsurgical inflammation than the oral route. Regarding the dose, dexamethasone at 4 mg presents a clinical effect like that at 8 mg, which is why dexamethasone at 4 mg was used intramuscularly in this study.

It is of great importance to mention that some years ago, experimental preclinical studies began to be carried out on the association of a corticosteroid (dexamethasone) with B vitamins, and synergistic analgesic, anti-inflammatory, and antiallodynic effects were found, without increasing the incidence of adverse effects. Dexamethasone attenuates ectopic neuronal discharges in experimental neuromas, thus demonstrating its antiallodynic activity. The combination of dexamethasone with vitamin B12 promotes the production of brain-derived neurotrophic factor and the proliferation of Schwann cells in experimental animal models, producing analgesia and the regeneration of nerve fibers [10].

Below is a summary of the results obtained from the study of several articles where corticosteroids were used in patients between the ages of 16 and 65 undergoing oral or maxillofacial surgery (Table 6, Table 7, Table 8, Table 9 and Table 10).

6. Study the adverse reactions of glucocorticoids after prolonged use in patients undergoing oral and maxillofacial surgery.

The toxicity that occurs with the use of glucocorticoids is based on the average dose and the length of time for which they are administered. Likewise, it is necessary to know some of the contraindications: hypersensitivity to any of the components, simultaneous administration with live or attenuated vaccines, systemic fungal infection, osteoporosis, uncontrolled hyperglycemia, diabetes mellitus, uncontrolled hypertension, keratitis, and chickenpox infection [39].

Adverse effects are more frequent with higher doses, chronic use, and a prolonged period of use, although this is not necessarily always the case. Sequelae after their use are observed in up to 90% of patients who take them for more than sixty days.

Synthetic corticosteroids, such as prednisone, methylprednisolone, dexamethasone, and betamethasone, are typically more prone to cushingoid features, referring to the weight gain and fat redistribution observed with too much cortisol. This feature may progress within the first two months of glucocorticoid therapy and result in the suppression of HPA (hypothalamic–pituitary–adrenal) axis function. In adults, other adverse effects associated with prolonged steroid use include dyslipidemia, cardiovascular effects, psychiatric disorders, immunosuppression, and gastrointestinal and dermatological events [53].

## 5. Discussion

After the analysis of the investigated articles, it can be determined that the findings revealed that the use of corticosteroid therapy, either pre- or postsurgically in patients who meet the favorable systemic conditions for its use, is considered safe and effective for pain management, edema, inflammation, and lockjaw in oral and maxillofacial surgery.

Studies in various populations show that considering the duration of administration, the route of administration, and the dose administered, the possible risks and side effects are limited. It should be noted that a recent study showed that the use of group B vitamins, administered intramuscularly as an adjuvant in corticosteroid therapy, showed an increase in the analgesic effect of a glucocorticoid in the population studied [10].

For decades, researchers have investigated the feasibility of the continued use of glucocorticoids and have found contrasting results regarding their safety. Likewise, regarding the pharmaceutical route of use, the intramuscular route of administration is one of the best choices since it has been proven to be more effective in reducing postsurgical pain and inflammation than the oral route, which is in contrast to other authors, who state that the oral route is their preferred alternative [10].

When we refer to the pathologies caused by cervicofacial infections, where the use of corticosteroids as an adjuvant to antibiotic therapy has begun to yield positive results, it is of great importance to know that, to carry out safe treatment and seek the best results, glucocorticoids should not be administered alone but rather always be accompanied by an antibiotic [35].

New trends are currently emerging, and these have identified a gene that generates the benefits of steroids without the appearance of side effects. Scientists have found that once the KLF15 gene (Krüppel-like factor 15) is activated, glucocorticoids frequently indicate improving muscular resistance and alleviating muscular dystrophy. This advance is especially important for diseases causing progressive muscle loss [54].

### 5.1. Limitations

The following are the limitations found when carrying out this systematic review:

There are not enough articles that describe the effectiveness of the use of glucocorticoid therapy in populations between 16 and 65 years of age, either female or male.

Systematic reviews need to be updated.

Investigations into the newly emerging trends of therapies with glucocorticoids and adjuvants need to be carried out.

### 5.2. Future Perspectives

The KLF15 transcription factor (Krüppel-like factor 15) is stimulated by glucagon and glucocorticoids, with this impulse occurring in the fasting state. However, insulin negatively regulates the expression of KLF15 under feeding conditions [54].

Skeletal muscle proactively regulates systemic nutrient homeostasis through transcriptional adjustments in response to physiological signals. These gene regulatory pathways are somewhat remarkable in their ability to serve in the treatment of metabolic diseases. Upon characterizing the KLF15 cistroma in vivo in skeletal muscle, it was evidenced that most of the binding of KLF15 takes place in distal intergenic regions and is linked to genes related to the circadian rhythm and lipid metabolism. We also identified the critical interdependence between KLF15 and the peroxisome-proliferator-activated receptors (PPARδ) nuclear receptor in the regulation of lipid metabolic gene programs. Furthermore, it was shown that KLF15 and PPARδ are genome-wide, physically interrelated, and require each other to exert their transcriptional effects on target genes. This evidence indicates that KLF15 plays a vital role in metabolic adaptation through its activism on target genes and interactions with other nodal transcription factors such as PPARδ [54].

Inflammatory conditions, fibrosis, obesity, cardiovascular diseases, and cancer can be altered by factor KLF15. Knowing this information can aid in establishing the hidden molecular mechanisms behind the instigation of the gene transcription regulated by fasting, e.g., hepcidin’s role in the CRBN-KLF15 signaling pathway in hepatocytes [55].

## 6. Conclusions

Even though the information on the administration of corticosteroids is quite limited and, at the same time, somewhat controversial, it is necessary to recognize the success that most have achieved in assessments of their efficacy and high performance against inflammatory and immunological responses (even in cases of medical emergencies) in surgery, oral and maxillofacial surgery, and other health sciences in general.

There is still profound uncertainty that surrounds health professionals today when trying to determine whether the adverse effects of the use of steroids could endanger the patient’s life or whether their use would provide benefits due to their anti-inflammatory and immunomodulatory properties when used in their proper doses.

Studies have shown their ability to reduce morbidity, as well as the adverse effects that their prolonged use without a basis entails. It can be said that, for the most part, researchers promote the use of steroids, using the appropriate route of administration for each patient, as preventive, postoperative, or even maintenance therapy to prevent the recurrence or worsening of the injury or condition. In a certain way, due to certain systemic conditions in certain patients, the use of these hormones is a risk and is contraindicated, and alternative therapies that can be carried out with the greatest possible efficiency should be sought.

The studies that were analyzed for cases of oral and maxillofacial surgeries mostly used dexamethasone (4–8 mg) as the main drug of choice, suggesting that the intramuscular route is more effective when seeking to attenuate unwanted clinical manifestations, such as facial edema, lockjaw, inflammation, and pain resulting from the intervention.

For patients with cervicofacial infections, the use of high doses of corticosteroids (hydrocortisone (200 to 1050 mg); dexamethasone (8 to 10 mg); methylprednisolone (1 to 3 mg/kg), intravenously, in a short period of time at the same time as the administration of antibiotic therapy may be beneficial.

Regarding the population between 10 and 16 years of age, no studies were found that could determine or provide clarity on whether the use of glucocorticoids is effective and safe in major surgeries of the oral cavity, facial skeleton, and cervical structures in this population.

It should be noted that, in the population of adolescents and young adults in an age range of (10–16) or (18–35), postsurgical manifestations are subject to the duration of the surgery and whether an osteotomy is performed.

It is vitally important to understand that once corticosteroid therapy has started, it cannot be stopped abruptly since the production of cortisol managed by the adrenal glands is diminished or practically stopped, which implies that the use of corticosteroids must be gradually withdrawn until the glands can once again begin to produce cortisol on their own.

The use of these drugs provides the advantage that they can be used alone or combined with other medications for optimization and better results in terms of disease control and patient recovery. Nevertheless, it must be known that the interaction of glucocorticoids with certain medications, such as Ritonavir, can lead to possible toxicity and cause iatrogenic Cushing’s syndrome.

## Figures and Tables

**Table 1:** Summary of the selection criteria.

Inclusion Criteria	Exclusion Criteria
Respond to the objectives set.	Not related to the objectives.
Study types: observational, randomized clinical trials.	Full texts not available.
Articles published in the last 10–15 years.	Articles that do not meet the inclusion criteria or with little proven scientific evidence (PEDro scale less than 7).
Articles are open access, free, and complete.	
Articles published in English, Spanish, Portuguese.	
Population between 10 and 65 years old/any race and sex.	
Valued with the PEDro scale higher than 7.	

**Table 2:** Cochrane risk of bias tool to assess the methodologies of the examined studies. Low risk (+), high risk (-) uncertainty (?).

1st Author, Year	Bias from Random Sequence	Bias Allocation Concealment	Bias Blinding Participant and Personnel	Bias Blinding Outcome	Incomplete Outcome Data	Selective Report	Other Bias
Bhandage 2018 [7]	+	+	?	+	+	+	+
De la Cruz Carranza 2013 [8]	+	+	-	+	+	+	+
Manriquez-Guzman 2013 [9]	+	+	-	+	+	+	+
Chavez-Rimache 2020 [10]	+	+	+	+	+	+	+
Nunez-Dias 2019 [11]	+	+	+	+	+	+	+

**Table 3:** Summary of the main drug pathologies and routes of administration in oral and maxillofacial surgery.

Pathology	Drug	Route of Administration
Temporomandibular joint disorders.	10 mg/day methylprednisolone, betamethasone acetate, triamcinolone acetonide, and hydrocortisone.	Intra-articular route. Limit application to 4 times a year.
Oral lichen planus.	Triamcinolone acetonide 0.1%, betamethasone sodium phosphate 0.1–0.05%, clobetasol 0.05%.	Topical route. Two or three times a day if a lesion is present.
Bell’s palsy.	Prednisolone 1 mg/kg/day	Orally. 7–10 days.
Recurrent aphthous stomatitis.	Hydrocortisone hemisuccinate 2.5 mg, triamcinolone acetonide 0.1%, dexamethasone 0.5 mg.	Topical route on the lesion two or three times a day and before sleeping (gel or paste).
Behçet’s disease.	Immunosuppressive therapy. In acute phase, prednisone 40–60 mg/day.	Orally.
Pemphigus diseases.	Prednisone 0.5–1.5 mg/kg/day or 10–40 mg highly potent topical corticosteroids.	Orally and topically, reducing or increasing the dose according to the severity of the lesion.
Erythema multiforme.	Clobetasol propionate rinses in aqueous solution. In case of aggravation of the lesion, prednisone 40–80 mg/kg/day, methylprednisolone 1 mg/kg/day (3 days).	Topical route. Mouthwashes. Oral route in case of aggravation (1–2 weeks and reduce dose).
Postherpetic neuralgia.	Methylprednisolone 40 mg/10 day.	Intramuscular route.
Central giant-cell granuloma.	Dexamethasone 0.5% (3 mg/mL/0) injections once a week for six weeks.	Intramuscular route.

**Table 4:** Use of corticosteroids for temporomandibular joint (TMJ) conditions and other oral and maxillofacial injuries. The role of corticosteroids in today’s oral and maxillofacial surgery [2].

Drug	Usual Dose
Hydrocortisone (IV, IM, topical)	20 mg–240 mg/day
Prednisone (orally)	5 mg–60 mg/day
Prednisolone (orally)	5 mg–60 mg/day
Methylprednisolone (orally, IV, IM)	10 mg–0 mg/day
Triamcinolone (topical, orally)	10 mg–60 mg/day or 0.1 mg–0.3 mg
Betamethasone (IV, IM, orally)	0.6 mg–7.2 mg/day
Dexamethasone (IV, IM, orally)	0.75 mg–9.0 mg/day

**Table 5:** Interactions between glucocorticoids and other drugs.

Albendazole: GCs reduce the metabolism of antiparasitic agents, favoring gastrointestinal and hepatic toxicity.
Antacids: reduce GC absorption.
Antifungal azoles: increase plasma levels of GCs, causing adverse effects.
Barbiturates: reduce GC catabolism and reduce activation of the prodrugs prednisone and methylprednisolone.
Thyroid hormones: accelerate GC catabolism, which leads to loss of efficacy.
Progestogens and oral contraceptives: reduce GC catabolism increasing the effect and promoting toxicity.
GCs are CYP3A4 inducers, which can reduce the efficacy of some drugs by increasing their catabolism, e.g., benzodiazepines, tretinoin, quetiapine, statins, tyrosine kinase inhibitors.

**Table 6:** Summary of the results obtained in the article Bhandage et al. 2018 [7].

Article	Purpose of the Study	Intervention	Results	Conclusion
Evaluation of efficacy of peri-operative administration of hydrocortisone and dexamethasone in prevention of post-operative complication in oral and maxillofacial surgeries.	Examine the roles of intraoperative administration of hydrocortisone and postoperative dexamethasone in minimizing postoperative complications after major surgeries of the oral cavity under general anesthesia.	N = 20 patients (25–65 years). General anesthesia (intubation and extubating) was used. Procedures included maxillary, mandibular, and zygomatic-maxillary complex fractures (trauma) and surgeries to treat pathologies such as keratocystic odontogenic tumors.	Intervention: single IP dose of hydrocortisone. Result: 2nd postsurgical day: 70% pain reduction. Fourth postsurgical day pain reduction of 97% according to numerical scale (EN), accompanied by 12 mm reduction in edema. No patient developed ADRs, such as nausea or vomiting, at the postoperative level.	Administration of a single IP dose of hydrocortisone and adjusted postoperative dexamethasone helps combat most postoperative complications after surgical interventions; therefore, it is an effective and safe drug.
Bhandage et al. 2018 [7].				

**Table 7:** Summary of the results obtained in the article De la cruz Carranza et al. 2013 [8].

Article	Purpose of the Study	Intervention	Results	Conclusion
Effectiveness of 4 and 8 mg prophylactic dexamethasone to control post-surgical swelling of impacted third molars: A randomized parallel-group clinical trial.	To verify the effectiveness of prophylactic dexamethasone orally (PO) 8 mg with 4 mg to control postsurgery edema of impacted third molars.	N = 66 patients (18–30 years). Lower third molar included asymptomatic and moderate level of difficulty according to the classification of Koerner et al.	Intervention: 27 received 8 mg PO prophylactic dexamethasone and 27 4 mg. Result: 8 mg dexamethasone was more effective than 4 mg dexamethasone.	8 mg PO prophylactic dexamethasone is more effective than 4 mg for controlling postsurgery edema of third molars.
De la Cruz Carranza et al. 2013 [8].				

**Table 8:** Summary of the results obtained in the article Manriquez- Guzman et al. 2013 [9].

Article	Purpose of the Study	Intervention	Results	Conclusion
Glucocorticoids as a prophylactic anti-inflammatory in inferior third molar surgery.	To administer glucocorticoid medication (dexamethasone 8 mg via IM) 1 h before the treatment of complex exodontia via intramuscular route in one group and not to the other group, later evaluating the presence of severe acute inflammation.	N = 116 patients (21–45 years female/male). Randomly divided into two groups, only one received glucocorticoid medication before treatment.	Intervention: dose of dexamethasone 8 mg IM one hour before treatment. Result: it was found that 92% of the group that did not receive previous medication presented acute pain during the first 48 h, and 82% and 80% presented signs of edema and trismus, respectively. In contrast, 12%, 4%, and 2% of patients who received prior medication presented signs and symptoms of acute pain, edema, and lockjaw, respectively.	The appearance of signs and symptoms of severe acute inflammation was greater in the group that did not receive glucocorticoid medication before the intervention.
Manrique-Guzmán et al. 2013 [9].				

**Table 9:** Summary of the results obtained in the article Chavez-Rimache et al. 2020 [10].

Article	Purpose of the Study	Intervention	Results	Conclusion
Anti-inflammatory effect of dexamethasone and B vitamins in third molar surgery. Randomized clinical trial.	To analyze the anti-inflammatory effect of the preoperative administration of the combination of dexamethasone with B vitamins in mandibular third molar surgeries.	N = 54 patients (18–25 years). Control group was administered 4 mg of dexamethasone and the experimental group the combination of 4 mg of dexamethasone with vitamins B1, B6, and B12: via IM before surgery. Pain was evaluated using the visual analogue scale (VAS).	It was shown that the greatest magnitude of pain appeared at 24 h, being significantly lower in the experimental group. Facial swelling increased progressively until the 3rd day, with no significant difference between the groups.	A significantly greater analgesic activity and a significantly lower total consumption of analgesics were evidenced in the group that used dexamethasone and group B vitamins as an adjuvant.
Chávez-Rimache et al. 2020 [10].				

**Table 10:** Summary of the results obtained in the article Nunez-Diaz et al. 2019 [11].

Article	Purpose of the Study	Intervention	Results	Conclusion
Comparison of the anti-inflammatory effectiveness of dexamethasone as pre-surgical and post-surgical therapy in mandibular third molar surgery: A randomized clinical trial.	To compare the anti-inflammatory effectiveness of dexamethasone as pre-surgical and postsurgical therapy in mandibular third molar surgery.	N = 60 patients (16 to 35 years). Mandibular third molar extraction. Group A received 4 mg of dexamethasone intramuscularly pre-surgery, and group B received the same medication postsurgery. Pain intensity was evaluated using the numerical scale (EN).	The values of facial edema were lower in group A at 60 min than in group B. Regarding pain, the highest intensity was perceived at 6 h in both groups, with no significant difference between them.	Preoperative administration of dexamethasone produced a significantly greater reduction in facial edema following mandibular third molar surgery.
Nunez-Diaz et al. 2019 [11].				

## Data Availability

Not applicable.

## Abbreviations

KLF15: (Krüppel-like factor 15), GR: glucocorticoid receptor, MR: mineralocorticoid receptor, CRH: corticotropin-releasing hormone, ACTH: adrenocorticotropic hormone, IL: interleukin, TNF: tumor necrosis factor, IFNY: interferon gamma, NK: natural killer, NSAIDs: nonsteroidal anti-inflammatory drugs, VAS: visual analogue scale, HIV: human immunodeficiency virus, AI-GI: Guillain–Barré Syndrome, CPY3A4: cytochrome P450 3A4, TMJ: temporomandibular joint disorder, PLO: oral lichen planus, CGCG: central giant-cell granuloma, IV: intravenous, IM: intramuscular, SC: subcutaneous, IP: intraperitoneal, PLA2: phospholipase A2, INOS: nitric oxide synthase, HTA: arterial hypertension, GC: glucocorticoid, RAM: adverse drug reaction, EN: numerical scale, PTA: peritonsillar abscess, DNSI (deep neck space infection).
